# A Comparison of Methods to Maintain the Equine Cecal Microbial Environment In Vitro Utilizing Cecal and Fecal Material

**DOI:** 10.3390/ani12152009

**Published:** 2022-08-08

**Authors:** Jennifer L. MacNicol, Simone Renwick, Caroline M. Ganobis, Emma Allen-Vercoe, Jeffery Scott Weese, Wendy Pearson

**Affiliations:** 1 Department of Animal Biosciences, Ontario Agricultural College, University of Guelph, Guelph, ON N1G 2W1, Canada; 2 Department of Molecular and Cellular Biology, College of Biological Sciences, University of Guelph, Guelph, ON N1G 2W1, Canada; 3 Department of Pathobiology, Ontario Veterinary College, University of Guelph, Guelph, ON N1G 2W1, Canada

**Keywords:** horse, microbiome, metabolome, gastrointestinal, cecum

## Abstract

**Simple Summary:**

In vitro systems for the fermentation of equine gastrointestinal (GI) content provide researchers with the ability to evaluate changes which may occur due to external influences but which cannot be accessed in vivo. The objective of this study was to evaluate three fermentation systems to replicate the equine cecal environment with regard to the microbiome and metabolite profile. The microbiome and metabolome of the fecal slurry used as inocula in this study were not representative of the cecal systems and care should be taken if feces are to be used to mimic proximal hindgut regions such as the cecum. However, the microbiome of the cecal inoculum maintained in either a chemostat batch fermenter or anaerobic chamber was fairly comparable. The metabolite concentrations, but not rate of production, were significantly different between the two cecal systems. These results provide a context to determine the most appropriate methods by which to create a fermentation system to reflect the equine cecal environment. They also highlight that caution must be exercised as many factors may influence the microbial and metabolic profiles within these systems; as such, they can best be used to demonstrate trends and gross reactions to environmental stimuli.

**Abstract:**

The equine gastrointestinal (GI) microbiota is intimately related to the horse. The objective of the current study was to evaluate the microbiome and metabolome of cecal inoculum maintained in an anaerobic chamber or chemostat batch fermenter, as well as the fecal slurry maintained in an anaerobic chamber over 48 h. Cecal and fecal content were collected from healthy adult horses immediately upon death. Cecal fluid was used to inoculate chemostat vessels (chemostat cecal, *n* = 11) and vessels containing cecal fluid (anaerobic cecal, *n* = 15) or 5% fecal slurry (anaerobic fecal, *n* = 6) were maintained in an anaerobic chamber. Sampling for microbiome and metabolome analysis was performed at vessel establishment (0 h), and after 24 h and 48 h of fermentation. Illumina sequencing was performed, and metabolites were identified via nuclear magnetic resonance (NMR). Alpha and beta diversity indices, as well as individual metabolite concentrations and metabolite regression equations, were analyzed and compared between groups and over time. No differences were evident between alpha or beta diversity in cecal fluid maintained in either an anaerobic chamber or chemostat. The microbiome of the fecal inoculum maintained anaerobically shifted over 48 h and was not comparable to that of the cecal inoculum. Metabolite concentrations were consistently highest in chemostat vessels and lowest in anaerobic fecal vessels. Interestingly, the rate of metabolite change in anaerobic cecal and chemostat cecal vessels was comparable. In conclusion, maintaining an equine cecal inoculum in either an anaerobic chamber or chemostat vessel for 48 h is comparable in terms of the microbiome. However, the microbiome and metabolome of fecal material is not comparable with a cecal inoculum. Future research is required to better understand the factors that influence the level of microbial activity in vitro, particularly when microbiome data identify analogous communities.

## 1. Introduction

As hindgut fermenters, horses have evolved in such a way that they can derive the majority of their energy from the microbial fermentation of a highly fibrous diet, which ideally consists mainly of grass and hay. Horses can obtain up to 70% of their energy requirements from volatile fatty acids (VFAs) produced in the hindgut via microbial fermentation of fiber when fiber represents their major energy source [1]. However, as monogastric animals, digestion and nutrient absorption through the small intestine is still a critical means of obtaining energy for horses. In many modern equine diets, particularly of sport horses, concentrates provide significant energy via enzymatic digestion. The cecum, which sits at the beginning of the hindgut, is a large, blind-ended sac that acts by mixing incoming content from the small intestine with symbiotic microbes that reside within the cecal lumen. Volatile fatty acid production in the cecum accounts for approximately 30% of the digestible energy in the horse in long-stem fiber-based diets [2]. As such, the function and activity of the cecum are of significant interest. However, the cecum is difficult to access in vivo. Cecal cannulation is an option for in vivo sampling, but it is a technically challenging surgical procedure that is not always well tolerated in equines [3,4,5,6]. For these reasons, the development of robust in vitro methods provide a means to explore the dynamics of the unique microbial ecosystems within the equine hindgut.

The use of in vitro fermentation represents a practical alternative to in vivo experimentation, particularly as a preliminary tool to establish areas that warrant further study and enable targeted in vivo follow up. In vitro studies, used to reflect the microenvironment of various GI departments, are a common technique. Such systems have been perfected for cecal content from poultry, swine, and in particular, the bovine ruminal environment, but are less evident when investigating the equine literature. In vitro fermentation of ruminal contents from cattle and cecal content from poultry is a common means for screening different feedstuffs. Frequently, filtered inoculum is diluted and mixed with the substrate under investigation, then left to incubate at 37–39 °C for 24 h under anaerobic conditions, occasionally with periodic sampling [7,8,9,10]. Chemical evaluation of the VFA profile following fermentation provides insight into the potential nutritional value of the dietary additives or feedstuffs under investigation. In equine nutrition, a fecal slurry is often used as an inoculum for in vitro digestibility trials [11,12,13,14]. Although fecal material is used as an indicator of hindgut function, deviations in the mechanical activity [15], VFA production and absorption [16,17,18], water balance [17], and microbial ecosystems [19,20,21] between hindgut compartments suggests that feces may not appropriately reflect hindgut microbial ecology and activity. In particular, feces are not likely a suitable substitute for samples from more proximal hindgut regions.

In vitro systems are much less commonly used to investigate the dynamics and activity of GI microbes in equine physiology. These systems present a powerful means of identifying potential pathological changes in the unique GI microbial ecosystems which exist within the horse. However, at present, there is limited information on how these systems react in various environments or which methods are best suited to a desired research question.

Continued investigation into the differences in microbial composition and activity between hindgut compartments is integral to generate context for in vitro results that utilize different inoculum sources. Limited exploration has been carried out into the effects of different fermentation methods to maintain equine hindgut microbial populations in vitro and potentially prolong the fermentation period. A greater understanding of how in vitro conditions impact the microbial ecosystem, and its activity, will aide in the selection and application of the most appropriate methods depending upon the desired aim of the research. These critical aspects of in vitro fermentation research require careful consideration. The use of modern techniques and the combination of multiomics datasets are necessary to continually advance this vital type of research. The objectives of this study were to evaluate and compare the microbial and metabolic profile of equine cecal fluid maintained for 48 h in either a simple anaerobic chamber or a chemostat batch fermenter, as well as equine fecal slurry maintained for 48 h in an anaerobic chamber.

## 2. Materials and Methods

### 2.1. Sample Collection

All sample material was collected from adult horses deemed healthy for consumption at Viande Richileu (QC, Canada). Horses were shipped to the abattoir from various facilities and locations. Therefore, long-term or short-term dietary information was not available. Although the time from last feed was not standardized subjectively, all collections were made from GITs which were full of luminal content consisting of only fibrous material. Any access to feed the horses had in transit would consist of long-stem fiber and no feed was available to horses upon arrival at Viande Richileu. Gastrointestinal tracts were collected within 10 min of death. The cecum was opened at the base, where the pH was measured using a portable pH meter (ST20, OHAUS, OHAUS corporation, NJ, USA). Following pH measurements, content was collected directly from the cecum, put into sterile containers, and placed on ice. When fecal material was also collected, it was taken directly from the lower rectum, put into sterile containers, and placed on ice. Cecal pH was measured at the time of collection using a portable pH meter. Containers of cecal and fecal material were placed in anaerobic jars with anaerobe packets (Mitsubishi AnaeroPack, Thermo Scientific). Jars were placed on ice and immediately transported from Viande Richileu (QC, Canada) to the University of Guelph (ON, Canada; approx. 9h). Aliquots of cecal fluid were serially centrifuged, and the supernatant was passed through a 0.22 uM filter and stored at −80 °C for future metabolite analysis. Fecal material underwent the same process, with the exception of the collection and vessel establishment samples (0 h), which were first diluted at 25% *wt/vol* [22] with sterile phosphate-buffered saline (PBS) and vigorously mixed prior to centrifugation.

### 2.2. Chemostat and Anaerobic Vessel Establishment and Sampling

Dual-vessel, single-stage chemostat bioreactors (Infors, Switzerland) were utilized as batch fermenters (chemostat cecal, *n* = 11). All vessels, tubing, and associated materials were sterilized prior to experiment initiation. Vessels were inoculated with 500 mL of cecal content within 24 h of collection (approx. 18 h following collection) in a laminar flow hood cleaned with 70% isopropyl alcohol and exposed to UV radiation for 15 min. The vessel pH was set to the cecal pH measurements taken upon sample collection and maintained using 5% NaOH and 5% HCl. Vessels were kept at 37 °C under anaerobic conditions using nitrogen gas and continually agitated via mechanical stirring.

An anaerobic chamber (Bactron IV, Sheldon Manufacturing, Cornelius, OR, USA) was utilized to maintain a 37 °C temperature and anaerobic conditions during vessel preparation and sampling. The anaerobic chamber was cleaned with 70% isopropyl alcohol prior to any sample manipulation. One hundred ml of cecal fluid was measured in sterilized graduated cylinders and used to inoculate sterilized 150 mL Pyrex jars (anaerobic cecal, *n* = 15). Initial collections only sampled cecal fluid and established chemostat and anaerobic chamber vessels. However, fecal material was also collected during two trips when the experimental design was adapted to adjust for equipment shortages. As chemostat equipment was unavailable at these times, the cecal fluid and fecal material were compared within the anaerobic chamber. For this reason, the number of samples for each method slightly differs. A 5% fecal slurry was made with 100 mL of sterilized PBS in a sterilized 150 mL Pyrex (anaerobic fecal, *n* = 6). Jars of inoculum were maintained in the anaerobic chamber on a plate shaker.

In chemostat vessels, 6 mL of content was removed directly from the vessel via sterile syringe. In anaerobic chamber vessels, 4 mL of content was removed directly from the vessel via sterile syringe. Content was removed every 12 h and the volume was replaced with sterile H_2_O. In anaerobic chamber vessels, the pH was also measured with a portable pH meter (ST20, OHAUS, OHAUS corporation, NJ, USA), which was disinfected using 70% isopropyl alcohol.

Subsamples for microbiome analysis were fully submerged in anhydrous EtOH for sample stabilization [23,24,25]. Subsamples used for metabolome analysis were treated as previously described. Subsamples were stored at −80 °C until further analysis of the 0 h, 24 h, and 48 h samples.

### 2.3. Microbiome and Metabolite Sample and Data Preparation

Samples for microbiome analysis were thawed and bacterial DNA was extracted using the E.Z.N.A Stool DNA Kit (Omega Bio-Tek, GA, USA) according to the manufacturer’s instructions. PCR amplification of the V4 region of the 16S rRNA gene was performed using the KAPA2 Fast HotStart ReadyMix (Sigma-Aldrich, Canada) and 515-F and 806-R modified primers [26]. PCR products were visualized using 5% agarose gel electrophoresis, then purified using Mag-Bind RXNPure Plus (Omega Bio-Tek, GA, USA) magnetic beads. A second PCR reaction was used for the attachment of Illumina adapter primers, and samples were repurified and submitted to the Agriculture and Food Labs at the University of Guelph for sequencing on an Illumina MiSeq.

Forward and reverse sequence reads were aligned and underwent a standard series of quality control filtering steps using mothur 1.44.3 [27,28] to remove primers, sequences that were of abnormal length, contained ambiguous base calls, or were not consistent with the V4 region, and chimeras. Sequences were assembled into OTUs using a de novo (open OTU) picking approach based on 97% similarity. OTUs were classified against the Ribosomal Database Project Classifier [29]. Random subsampling was performed to normalize the sequence count. Alpha diversity indices including richness (Chao1), evenness (Shannon evenness index), and diversity (inverse Simpson index) were calculated. Beta diversity indices including community membership (Jaccard index) and structure (Bray–Curtis index) were also calculated.

Filtered supernatant samples for metabolite analysis were standardized through the addition of a DSS-d6 Chenomx Internal Standard (Chenomx, EDM, CAN). Samples were read on a nuclear magnetic resonance (NMR) spectrometer with an operating field of ≥600 MHz using the METNOESY sequence [22] at the University of Guelph Advanced Analysis Center. Sample pH was measured using Cytiva Whatman wide-range pH indicator strips (Fisher Scientific, Canada). Spectral profiling and processing was performed using Chenomx software [22]. The metabolite concentrations from fecal samples diluted to 25% *wt/vol* were adjusted to match the dilution of the fecal slurry used as the inoculum.

### 2.4. Statistical Analysis

All statistical analyses were run in SAS 9.4 (SAS Inc., NC, USA) using a repeated measures analysis of variance (RM ANOVA) in PROC GLIMMIX. Statistical models, and fixed, random, and repeated effects were identified for each measurement analyzed (see below). Residuals were analyzed to identify the most appropriate covariance structure, and means of sample types, methods, times, and interactions were compared when appropriate. Interactions were sliced by type and time and Tukey adjustments were applied. *p* ≤ 0.05 was considered significant.

*pH*

Statistical analysis of the *pH* (γ) from anaerobic chamber vessels was calculated according to the following model:

γ_ijk_ = µ + ani_i_ + type_j_ + time_k_ + type ∗ time_jk_ + ε

where µ = the overall mean, ani_i_ = the random effect of animal (i = 1 to *n*), type_j_ = the fixed effect of sample type (j = cecal fluid, feces), time_k_ = the repeated fixed effect of time (k = 0, 12, 24, 36, 48 h), and the interaction between sample type and time, ε = the residual error.

### 2.5. Microbiome Analysis

Statistical analysis of alpha diversity indices and taxonomic relative abundance data for all phyla, orders, and genera with a total relative abundance greater than 1% (γ) were performed according to the following model:

γ_ijk_ = µ + ani_i_ + method_i_ + time_k_ + method ∗ time_jk_ + ε

where µ = the overall mean, ani_i_ = the random effect of animal (i = 1 to *n*), method_j_ = the fixed effect of fermentation method (j = chemostat cecal, anaerobic cecal, anaerobic fecal), time_k_ = the repeated fixed effect of time (k = 0 h, 24 h, 48 h), and for the interaction between fermentation method and time, ε = the residual error. For taxonomic data, a false discovery rate adjustment was applied to the *p*-values using a Benjamini–Hochberg correction for each taxonomic level within a comparison using the PROC MULTTEST procedure in SAS 9.4. An adjusted *p* < 0.05 was considered significant.

Beta diversity measures within the fermentation method over time, as well as between fermentation methods at each time, were evaluated with an analysis of molecular variance (AMOVA) and homogeneity of molecular variance (HOMOVA). Samples were also separated by sample type (cecal or fecal) and beta diversity indices were compared with AMOVA and HOMOVA.

### 2.6. Metabolite Analysis

Statistical analysis of individual metabolites (γ) was performed according to the same model as was used for alpha diversity indices, with the exception of the inclusion of the covariate slope.

In order to analyze the change over time in metabolite production and the potential differences between fermentation methods, a comparison of regression responses was performed for acetate, propionate, and butyrate. The variance partitions used in the ANOVA were kept and a dummy variable was used for the quantitative independent variable of time to further partition it into linear and quadratic terms. As the quadratic time term was significant, the solutions for each term of the quadratic equations for metabolite concentration were calculated for each fermentation method. Contrast statements were used to compare each of the regression components (intercept, linear term, and quadratic term) between each fermentation method.

## 3. Results

### 3.1. pH

**Figure 1:**
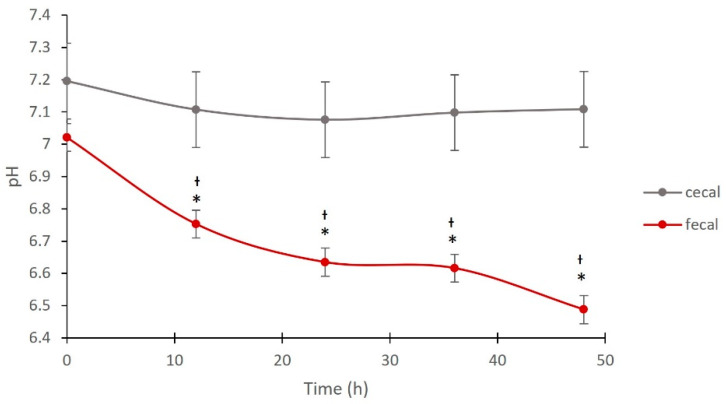
Comparison of pH between cecal and fecal vessels maintained in an anaerobic chamber. pH of cecal and fecal inocula maintained in an anaerobic chamber at 12 h intervals following vessel establishment (0 h). To inoculate 100 mL vessels, 100 mL of cecal fluid (anaerobic cecal, *n* = 15) or 5% fecal slurry (anaerobic fecal, *n* = 6) was used; * represents a significant difference between a timepoint and 0 h within a fermentation method (*p* < 0.05); † represents a significant difference between methods at a particular timepoint (*p* < 0.05).

The pH within anaerobic cecal vessels was consistent over the 48 h period. Within anaerobic fecal vessels, the pH continually decreased with the exception of the 24–36 h period. The pH was not significantly different between anaerobic cecal and anaerobic fecal samples at 0 h, but was different between the two sample types from 12 h onwards (Figure 1).

### 3.2. Microbiome

#### 3.2.1. Sequence Analysis

The total number of raw sequences was 19,999,401. Following quality control filtering, the total number of sequences was 15,288,548 (average per sample: 135,297; SD: 17,591; median: 132,918; range: 96,744–189,757). Sequences were subsampled at a sequencing depth of 90,000 sequences per sample to adjust for the uneven depth across samples. This resulted in a coverage of >99% for all samples.

#### 3.2.2. Alpha Diversity

**Figure 2:**
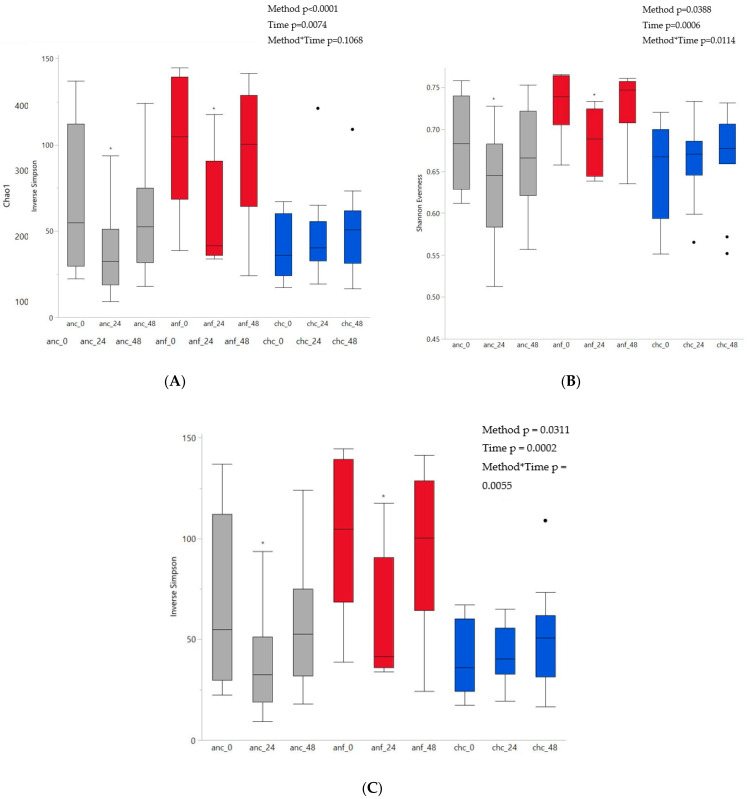
Comparison of alpha diversity indices. (**A**) Richness (Chao1); (**B**) evenness (Shannon evenness); (**C**) diversity (inverse Simpson), within fermentation methods over time. To inoculate 100 mL vessels maintained in an anaerobic chamber, 100 mL of cecal fluid (anc, *n* = 15, grey) or 5% fecal slurry (anf, *n* = 6, red) was used. To inoculate chemostat vessels maintained as batch fermenters (chc, *n* = 11, blue), 500 mL of cecal fluid was used. * represents a significant difference from time 0 within a fermentation method (*p* < 0.05).

There was a significant effect of time (*p* = 0.0074) and fermentation method (*p* < 0.0001) on richness (Figure 2A). Richness was higher in anaerobic fecal samples than either anaerobic cecal (*p* < 0.0001) or chemostat cecal (*p* < 0.0001) samples, but richness in anaerobic cecal and chemostat cecal samples was not different (*p* = 0.7557). Richness decreased in anaerobic cecal samples by 24 h, and both anaerobic cecal as well as anaerobic fecal samples had decreased richness by 48 h. However, richness remained consistent in chemostat cecal samples over time. There was a significant interaction between fermentation method and time regarding evenness (*p* = 0.0114) and diversity (*p* = 0.0055) (Figure 2B,C, respectively). Within both anaerobic cecal and anaerobic fecal fermentation methods there were initial decreases in evenness and diversity between 0 h and 24 h, which increased back to vessel establishment by 48 h. No change in evenness or diversity over time was apparent in chemostat cecal samples. At the time of vessel establishment, evenness and diversity in anaerobic fecal samples were higher than anaerobic cecal and chemostat cecal samples. However, no difference in evenness or diversity between anaerobic cecal or chemostat cecal samples was noted at any time. Both evenness and diversity differed between anaerobic cecal and anaerobic fecal vessels at 48 h.

#### 3.2.3. Beta Diversity

**Figure 3:**
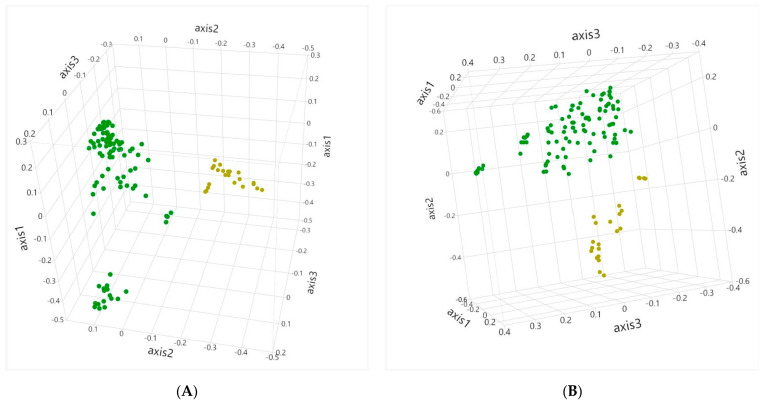
Beta diversity indices represented by 3D PCOA graphs. (**A**) Jaccard index and (**B**) Bray–Curtis index of cecal fluid samples (green) and fecal inoculum samples (yellow) maintained in vitro over 48 h.

There were no changes over time with regard to beta diversity within any of the fermentation methods. When an AMOVA was run on the Jaccard index and Bray–Curtis index, there were no differences at any time between anaerobic cecal and chemostat cecal samples. However, anaerobic cecal and chemostat cecal samples were both different from anaerobic fecal samples (*p* < 0.01) at all times. A HOMOVA analysis did not identify any differences between the Jaccard or Bray–Curtis index of any fermentation methods. When all timepoints were included in the analysis and samples were only separated by inoculum type (cecal fluid vs. feces), there was distinctive clustering by sample type upon visual inspection of both the Jaccard and Bray–Curtis PCOA graphs (Figure 3). There was also a difference between sample types for both the Jaccard index and Bray–Curtis index when analyzed by AMOVA (*p* < 0.001), and a difference between sample types for the Jaccard index when analyzed by HOMOVA (*p* = 0.02).

#### 3.2.4. Taxonomic Data

In order to best focus on our research question and evaluate the difference between fermentation methods, as well as the change within method over time, particular attention was given to differences between cecal fluid fermentation methods (anaerobic cecal and chemostat cecal) compared to fecal methods (anaerobic fecal); differences between the cecal methods (anaerobic cecal vs. chemostat cecal); and lastly, differences between the anaerobic chamber methods (anaerobic cecal and anaerobic fecal) and the internally stabilized batch fermenter method (chemostat cecal).

Selenomonadales, Subdivision5_unclassified, Bacteroidales, Desulfovibrionales, and Phascolarctobacterium were dissimilar between fecal and cecal inoculum samples at 0 h. The relative abundances of Proteobacteria, Enterobacteriales, and Lactobacillales changed over time in both cecal fermentation methods, but remained constant in fecal slurry samples. Between cecal fermentation methods the majority of differences were noted at 24 h. In all methods, Fusobacteria and Fusobacterium differed from 0 h at 24 h and 48 h in all methods. Bacteroides increased in all methods by 48 h. The full scope of these results are presented in Table 1. However, there were several sporadic variations that did not aide in resolving the objectives of this study (data not presented).

### 3.3. Metabolites

Metabolites identified in the majority of cecal and fecal inoculum samples using NMR included acetate, propionate, butyrate, isobutyrate, valerate, isovalerate, phenylacetate, and 3-phenylpropionate. At all times, the concentrations of these metabolites were significantly higher in cecal samples (anaerobic cecal and chemostat cecal) than in anaerobic fecal samples (Table 2). The concentration of acetate, butyrate, and isovalerate were significantly higher in chemostat cecal than anaerobic cecal samples at all times, and the concentration of propionate and valerate were higher in chemostat cecal than anaerobic cecal samples at 24 h and 48 h. In both anaerobic cecal and chemostat cecal samples, the concentrations of all metabolites increased by 24 h and remained higher than baseline at 48 h. This was also the case for anaerobic fecal samples regarding the concentration of acetate, butyrate, and propionate. In anaerobic fecal samples, isovalerate and valerate had significantly increased by 24 h and significantly increased again by 48 h, and isobutyrate significantly increased from baseline by 48 h, whereas 3-phenylpropionate and phenylacetate did not change. However, the concentrations of 3-phenylpropionate and phenylacetate were below the 0.03 mM range in anaerobic fecal vessels and, therefore, the absolute concentrations are less reliable.

**Figure 4:**
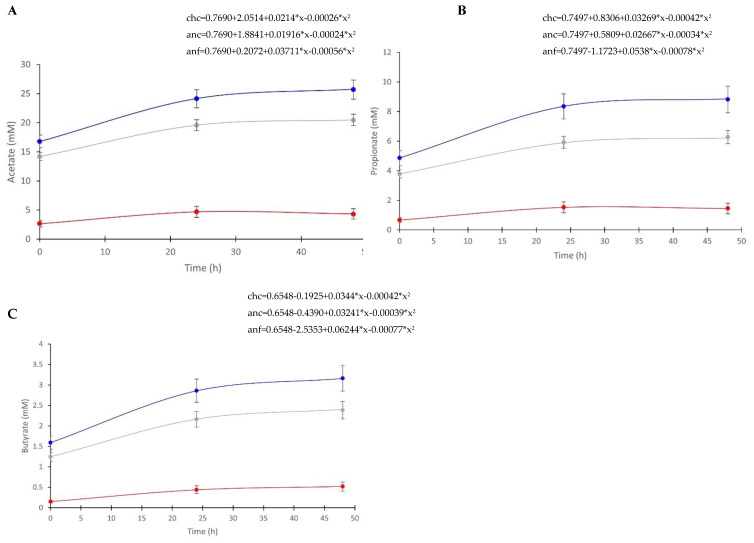
Regression analysis of metabolites. (**A**) Acetate; (**B**) propionate; (**C**) butyrate, as measured by NMR over time in inoculum maintained under different conditions. To inoculate 100 mL vessels maintained in an anaerobic chamber, 100 mL of cecal fluid (anc, *n* = 15; gray) or 5% fecal slurry (anf, *n* = 6; red) was used. To inoculate chemostat vessels maintained as batch fermenters (chc, *n* = 11; blue), 500 mL of cecal fluid was used.

Regression analysis identified a quadratic relationship between the concentration of acetate, butyrate, and propionate vs. time (Figure 4). These equations represent the rate of metabolite change, signifying the metabolite production over time in the various methods. The regression equations were different in all methods due to significantly different intercepts between the methods. However, the regression slopes were not different between any of the methods for acetate and propionate. The regression slope of butyrate was not different between anaerobic cecal and chemostat cecal samples, but was different between both anaerobic cecal and chemostat cecal compared to anaerobic fecal samples.

## 4. Discussion

Differences between all methods of in vitro fermentation were observed within this study. In particular, there was a notable distinction regarding both the microbial and metabolic parameters of cecal and fecal inocula. Fecal samples demonstrated higher richness, evenness, and diversity at the time of vessel establishment. Community membership and structure were also different between the two inoculum types and distinct clustering was evident upon visualizations with PCOA. These results are consistent with other studies that have demonstrated dissimilarities in the microbiomes of the different regions along the equine hindgut [19,20]. In general, rectal samples are not entirely representative of other regions, and caution is advisable when using fecal samples to assess other GIT compartments. Our results suggest that the use of a fecal slurry as an in vitro inoculum is not a suitable proxy for cecal fluid when evaluating the microbiome. The use of equine fecal inocula in batch culture was validated as an in vitro means of predicting gas production, digestibility, and the nutritive value of equine feeds [11,30]. Interestingly, we found the rate of change in metabolite production over time was not different between cecal and fecal samples for acetate and propionate, two major VFA by-products of microbial metabolism. One could speculate that the similar microbe activity observed within our study, despite the distinct microbial communities, is one factor which may contribute to the reliability of fecal inoculum when predicting feed digestibility for horses. However, as the focus of in vitro digestibility trials are measurements of nutrient breakdown, the microbial comparisons between inocula and the in vivo communities are of lesser consequence and generally left unaccounted for. Our results demonstrate how distinct microbial communities may be functionally similar. This concept of functional redundancy between communities [31] is, in part, why assessments that only account for microbial composition can be challenging to interpret. Global assessment of the microbial metabolic activity within in vitro systems is an essential component of developing a comprehensive picture of how the system behaves. Although the three major volatile fatty acids (acetate, propionate, butyrate) are often considered, other microbial metabolites play a role in demonstrating the activity of the system. Thus, the use of untargeted NMR represents a valuable tool to enrich our ability to evaluate and compare different in vitro systems. Nevertheless, it is important to take into consideration that despite the use of an untargeted NMR approach, the quantification of microbial metabolites was still based on a relatively narrow assessment of the entire metabolic profile within these samples. As such, our functional evaluation remains somewhat restricted and conveys chiefly a broad picture of microbial behavior.

Shifts in the relative abundances of particular taxa over time were evident within each system. Fluctuations in the relative abundance of individual taxa were anticipated. Although Bailey et al. [32] did not find changes in streptococci, lactobacilli, or Gram-negative anaerobes following the 24 h incubation of cecal inoculum, these were measured by CFU counting following culture. Although culture can provide meaningful data with regard to total counts of particular microbes, it lacks the ability of high-throughput sequencing to capture more extensive details regarding the taxonomic composition of a sample. Conversely, similar to our results, Biddle et al. [33] identified changes at the phyla and family level in samples of equine fecal material incubated over 48 h. Due to the community stability within all methods, it is possible that the practical relevance of these alterations in individual taxa with regard to the functioning of the microbial ecosystems is nominal. It is of note that there were substantial differences in certain taxa between individuals, with some individuals demonstrating a relative abundance of 0 for particular taxonomic groups, whereas other individuals had relative abundances as high as 30% for those same groups. This is not unexpected and aligns with results from other studies that observed substantial inter-individual variability within the GI microbiomes of horses [34,35,36,37]. However, despite these individual differences, our focus was the way in which communities and individual metabolic and microbiological parameters changed within individuals over time when different fermentation methods were used. Our focus was the comprehensive assessment of different systems, information which is currently lacking with regard to the fermentation of equine GI inoculum. Hence, individual differences between our subjects were less relevant compared to studies attempting to evaluate and generalize in vivo dietary or treatment interventions. Nevertheless, these considerable inter-individual taxonomic variations are an important consideration with regard to microbiome research and these data emphasize the necessity of finding ways to collect samples from several individuals. A large group of subjects is also important when the research question involves generalizing study results to a broad population.

Evaluations of how equine cecal or fecal taxa shift over time in vitro is lacking. However, taxonomic shifts are a critical component of the methodology when considering research regarding the microbiome. Changes in the relative abundance of the phylum Fusobacteria and the genus *Bacteroides* occurred over time, regardless of the fermentation method in this study. These results indicate there are particular taxa that will be preferentially affected over time when removed from their environment regardless of the inoculum source or the in vitro system employed. The changes in bacterial orders and genera that were identified in our study over time indicate that in vitro methods may not be an ideal approach when investigating responses to treatment of certain taxa, particularly at lower taxonomic classifications. In cases when the response of individual taxa is desired, prior justification that those specific microbes are stable in vitro is required. When taxa that are sensitive to in vitro conditions, such as *Fusobacterium* or *Bacteroides*, change in response to treatment in vitro, it is prudent to view these results critically as they may reflect the sensitivity of the particular bacteria to an in vitro environment as opposed to a true treatment effect. However, on a community scale, relatively stable membership and structure were evident over the entire 48 h in all fermentation methods. Therefore, the use of raw inocula obtained directly from the GIT can provide a generally consistent environment from which large shifts of the microbial population can be evaluated with more reliability than that of particular taxa.

The longer a system can be maintained, the more possibilities there are with regard to manipulation; therefore, the length of time the microbial community is stable is critical knowledge. However, without continuous flow systems, the community will eventually deteriorate as microbes continue to produce VFAs that are not removed and lack new sources of nutrients to metabolize. The quadratic relationship demonstrated over time between acetate, propionate, and butyrate within all systems, identified as an initial rapid production of metabolites followed by a plateau, is hard to interpret without microbial and metabolic data from timepoints beyond 48 h. The time course used in this study was based on previous in vitro studies that utilized equine [32,38], porcine [39,40], and poultry [7,8] cecal content, which was maintained in batch incubations for 24 h. Thus, 48 h provided a substantially longer timeline. Whether this metabolic plateau represents the beginning of an ecosystem crash, or a period of stabilization, requires a longer incubation period to determine. It is possible the cold storage of the inoculum prior to vessel establishment may have altered microbial activity or viability. Murray et al. [41] found that the use of frozen equine feces as a source of inoculum significantly reduced the extent and rate of substrate fermentation in vitro. Microbial lag can be the result of cold storage prior to culture, but it is not evident when bacteria are cooled in an environment where they are at maximal growth [42]. Although kept on ice and chilled, the cecal and fecal contents used as the source of inoculum in our study were never frozen. Furthermore, our results demonstrated an initial upsurge in metabolite generation as opposed to a delay.

The cecal microbiomes within the chemostat and anaerobic chamber were comparable throughout the 48 h period. Although measures of alpha diversity differed from vessel establishment at 24 h in anaerobic cecal samples, these measures were not different between anaerobic cecal and chemostat cecal methods at any time. Furthermore, no differences were apparent with regard to measures of beta diversity and no visual clustering was evident in PCOA between either method over time, indicating that the communities were fairly analogous throughout the experiment. Conversely, the concentrations of major microbial metabolites were consistently lower in anaerobic cecal compared to chemostat cecal samples, demonstrating a clear distinction between the level of microbial activity within the two methods. Conversely, the rates of change in acetate, propionate, and butyrate were equivalent between the two methods. Thus, it appears the microbes were acting in a similar manner in both systems, but at a lower level within the anaerobic chamber. The cause of the lower level of activity within the anaerobic cecal method is challenging to interpret. pH plays a substantial role in determining the growth [43], as does VFA production [44] of colonic microbes. Given that the pH within the cecal anaerobic chamber system was consistent over the 48 h period, it seems unlikely that microbe sensitivity to pH was a predominant factor impacting the production of metabolites therein. In chemostats, the pH was also stable as it was maintained by a pH sensor and automatic additions of NaOH and HCl. Therefore, the difference in the microbial level of activity between the two systems cannot be attributed to pH variability in either system. Similarly, both methods were maintained anaerobically at 37 °C and agitation was present within both systems. It is possible that the greater volume used in the chemostat system played a role by maintaining higher levels of microbe viability. In vitro inoculum volumes can range from 10 mL of 1:3000 diluted chicken cecal content [7,8] to 1320 mL of rumen fluid [45,46]. Although it appears inoculum volume is not often considered and its effect potentially deemed not relevant when results are standardized in terms of concentration, this may not be the case. Initial inoculum volume has been demonstrated to be an important condition with regard to the generation of biomass concentration, with greater initial volumes not resulting in proportionally greater biomass yields [47]. With regard to the fermentation of plant material, it appears that anaerobic digestion can be enhanced through the use of an increased inoculum volume [48]. Another possibility is that the bigger volume of inoculum used in the chemostat provided more raw substrate for the microbes present to metabolize. The inoculum was not strained in this study, as cecal bacteria attach to plant and forage cell walls [49], and we wished to capture a comprehensive spectrum of luminal microbes and their activity. Although the exact effect of inoculum volume on microbial activity is unclear at this time, it appears that greater consideration of inoculum volume should be made in future studies.

## 5. Conclusions

The microbiome profile of cecal fluid maintained in a batch fermenter with a conserved and stable internal environment is fairly consistent with cecal fluid simply maintained in an anaerobic chamber over a 48 h period. The metabolic profile of cecal fluid maintained in a batch fermenter compared to a more simplistic anaerobic chamber demonstrates the same rate of metabolite production, but a significantly higher level of activity. More research is required to determine if this is attributable to inoculum volume, a factor which may be more important with regard to the level of microbial activity than previously considered.

Cecal fluid used as an inoculum is not consistent with the microbial or metabolic profile of fecal slurry and care should be taken when extrapolating either microbial or metabolic results when fecal inoculum is used in place of cecal fluid in vitro.

## Figures and Tables

**Table 1:** Percent relative abundance of all phyla, order, and genera >1% that differ between inoculum types, cecal fluid fermentation methods, or between anaerobic chamber and chemostat methods.

		Method	Anaerobic Cecal			Anaerobic Fecal			Chemostat Cecal		
Time			0 h	24 h	48 h	0 h	24 h	48 h	0 h	24 h	48 h
% Relative abundance											
Phyla	Order	Genera									
Actinobacteria			0.54	1.31	1.28	0.33	0.71	0.71	0.90	1.02	1.32
Bacteroidetes											
	Bacteroidales		20.66	15.71	16.67	8.83	6.9	7.47	19.33	15.44	13.08
		*Bacteroides*	0.09	0.22	0.82	0.13	0.93	1.40	0.08	0.32	0.78
		*Prevotella*	2.63	1.60	1.39	0.69	0.56	0.35	1.87	0.99	0.33
		*Prevotellaceae_unclassified*	3.6	0.62	0.62	0.8	0.24	0.50	2.21	0.49	0.28
Firmicutes			34.18	38.03	39.88	29.36	38.68	43.77	38.2	40.66	43.97
	Lactobacillales		0.16	0.3	0.30	0.09	1.32	0.67	0.23	0.21	0.26
	Selenomonadales		3.04	4.26	3.61	1.65	4.00	3.17	4.28	2.76	2.56
		*Phascolarctobacterium*	2.63	3.55	3.03	1.23	2.33	2.38	3.52	2.33	2.21
	Clostridiales										
		*Lachnospiraceae_unclassified*	8.94	7.81	8.21	5.92	9.05	12.86	10.6	10.95	11.01
Fibrobacteria											
	Fibrobacterales		0.44	0.12	0.34	1.29	0.04	0.15	0.16	0.69	0.33
Fusobacteria			0.04	0.34	0.43	0.05	2.39	1.80	0.05 ^A^	0.22	0.26
Proteobacteria			3.11	6.17	3.78	1.77	7.95	3.45	3.59	2.54	2.6
	Enterobacterales		0.04	0.20	0.26	0.02	3.01	0.82	0.04	0.09	0.13
	Pasteurellales		0.07	0.16	0.10	0.01	0.54	0.12	0.07	0.07	0.05
Subdivision5											
	Subdivision5_unclassified		0.30	0.33	0.33	5.17	4.76	2.80	0.47	0.30	0.30
Spirochaetes											
	Spirochaetales		1.08	0.64	1.37	1.51	0.86	2.20	1.06	2.98	3.86
		*Treponema*	1.03	0.62	1.32	1.46	0.83	2.11	1.00	2.91	3.76
Verrucomicrobia											
	Verrucomicrobiales		3.95	5.42	2.37	1.61	5.98	2.75	5.12	2.54	2.12
		*Akkermansia*	3.79	5.29	2.3	0.26	0.66	0.44	4.98	2.46	2.05

To inoculate 100 mL vessels maintained in an anaerobic chamber, 100 mL of cecal fluid (anaerobic cecal, *n* = 15) or 5% fecal slurry (anaerobic fecal, *n* = 6) was used. To inoculate chemostat vessels maintained as batch fermenters (chemostat cecal, *n* = 11), 500 mL of cecal fluid was used.

**Table 2:** Metabolite concentration (mM) as measured by NMR over time in inoculum maintained under different fermentation methods.

Metabolite (mM)	Time (h)		
	0	24	48
Acetate			
anaerobic cecal	14.20 ± 0.7 *^a^*^A^	19.57 ± 0.9 *^b^*^A^	20.42 ± 1.0 *^b^*^A^
chemostat cecal	16.78 ± 1.1 *^a^*^B^	24.13 ± 1.6 *^b^*^B^	25.69 ± 1.7 *^b^*^B^
anaerobic fecal	2.65 ± 0.5 *^a^*^C^	4.67 ± 1.0 *^b^*^C^	4.3 ± 0.9 *^b^*^C^
Butyrate			
anaerobic cecal	1.25 ± 0.1 *^a^*^A^	2.16 ± 0.2 *^b^*^A^	2.38 ± 0.2 *^b^*^A^
chemostat cecal	1.59 ± 0.2 *^a^*^B^	2.86 ± 0.3 *^b^*^B^	3.16 ± 0.3 *^b^*^B^
anaerobic fecal	0.15 ± 0.03 *^a^*^C^	0.44 ± 0.10 *^b^*^C^	0.52 ± 0.11 *^b^*^C^
Propionate			
anaerobic cecal	3.78 ± 0.3 *^a^*^A^	5.91 ± 0.4 *^b^*^A^	6.3 ± 0.4 *^b^*^A^
chemostat cecal	4.86 ± 0.5 *^a^*^B^	8.35 ± 0.9 *^b^*^B^	8.82 ± 0.9 *^b^*^B^
anaerobic fecal	0.66 ± 0.2 *^a^*^C^	1.52 ± 0.4 *^b^*^C^	1.44 ± 0.3 *^b^*^C^
Valerate			
anaerobic cecal	0.327 ± 0.03 *^a^*^A^	0.560 ± 0.06 *^b^*^A^	0.581 ± 0.06 *^b^*^A^
chemostat cecal	0.410 ± 0.05 *^a^*^A^	0.753 ± 0.10 *^b^*^B^	0.814 ± 0.10 *^b^*^B^
anaerobic fecal	0.056 ± 0.02 *^a^*^B^	0.090 ± 0.03 *^b^*^C^	0.124 ± 0.05 *^c^*^C^
Isovalerate			
anaerobic cecal	0.294 ± 0.02 *^a^*^A^	0.501 ± 0.04 *^b^*^A^	0.539 ± 0.04 *^b^*^A^
chemostat cecal	0.374 ± 0.03 *^a^*^B^	0.636 ± 0.06 *^b^*^B^	0.678 ± 0.06 *^b^*^B^
anaerobic fecal	0.027 ± 0.009 *^a^*^C^	0.046 ± 0.02 *^b^*^C^	0.061 ± 0.02 *^c^*^C^
Isobutyrate			
anaerobic cecal	0.431 ± 0.04 *^a^*^A^	0.734 ± 0.07 *^b^*^A^	0.795 ± 0.07 *^b^*^A^
chemostat cecal	0.508 ± 0.05 *^a^*^A^	0.882 ± 0.08 *^b^*^A^	0.950 ± 0.09 *^b^*^A^
anaerobic fecal	0.051 ± 0.01 *^a^*^B^	0.056 ± 0.01 *^a^*^B^	0.091 ± 0.02 *^b^*^B^
Phenylacetate			
anaerobic cecal	0.147 ± 0.02 *^a^*^A^	0.264 ± 0.03 *^b^*^A^	0.265 ± 0.03 *^b^*^A^
chemostat cecal	0.197 ± 0.03 *^a^*^A^	0.308 ± 0.04 *^b^*^A^	0.329 ± 0.04 *^b^*^A^
anaerobic fecal	0.024 ± 0.006 *^a^*^B^	0.023 ± 0.006 *^a^*^B^	0.034 ± 0.008 *^a^*^B^
3-phenylpropionate			
anaerobic cecal	0.062 ± 0.01 *^a^*^A^	0.127 ± 0.02 *^b^*^A^	0.151 ± 0.02 *^b^*^A^
chemostat cecal	0.080 ± 0.02 *^a^*^A^	0.200 ± 0.04 *^b^*^A^	0.208 ± 0.04 *^b^*^A^
anaerobic fecal	0.017 ± 0.006 *^a^*^B^	0.018 ± 0.007 *^a^*^B^	0.019 ± 0.007 *^a^*^B^

To inoculate 100 mL vessels maintained in an anaerobic chamber, 100 mL of cecal fluid (anaerobic cecal, *n* = 15) or 5% fecal slurry (anaerobic fecal, *n* = 6) was used. To inoculate chemostat vessels maintained as batch fermenters (chemostat cecal, *n* = 11), 500 mL of cecal fluid was used; *^lowercase^* different lowercase superscripts represent significant differences within a row (*p* < 0.05); **^CAPITAL^** different capital superscripts represent significant differences of a metabolite within a column (*p* < 0.05).

## Data Availability

Data available in a publicly accessible repository The data presented in this study are openly available in NCBI SRA submission SUB11905104.
